# Speed, Change of Direction Speed, and Lower Body Power in Young Athletes and Nonathletes According to Maturity Stage

**DOI:** 10.3390/children9020242

**Published:** 2022-02-11

**Authors:** Mladen Živković, Nenad Stojiljković, Nebojša Trajković, Nikola Stojanović, Anđela Đošić, Vladimir Antić, Nemanja Stanković

**Affiliations:** Faculty of Sport and Physical Education, University of Nis, 18000 Nis, Serbia; snesadif@yahoo.com (N.S.); nele_trajce@yahoo.com (N.T.); nikola987_nish@hotmail.com (N.S.); andjela.djosic88@gmail.com (A.Đ.); vlada.antic@hotmail.com (V.A.); nemanjastankovic84@hotmail.com (N.S.)

**Keywords:** PHV, athletes, nonathletes, physical performance

## Abstract

The aim of this study was to establish the role of maturation on the development of physical performance in adolescent athletes and nonathletes. The total studied sample consisted of 231 participants (131 athletes: 72 boys with an average chronological age of 13.53 ± 0.7 and 59 girls with an average chronological age of 11.97 ± 0.8; 100 nonathletes: 47 boys with an average chronological age of 13.73 ± 0.47 and 53 girls with an average chronological age of 11.93 ± 0.33), distributed according to their biological maturity stage (Pre-, Mid-, and Post-Peak Height Velocity [PHV]) and to their gender. The assessment of physical performance was performed using the following tests: Countermovement jump (CMJ), countermovement jump with arm swing (CMJA), squat jump (SJ), five-jump test (5JT), 5 m sprint (5 m), 10 m sprint (10 m), 20 m sprint (20 m), T-test, Zig Zag, and Slalom. The differences in athletes according to biological maturity were identified in all variables except for 5 m (*p* = 0.33) and Slalom (*p* = 0.07), while in nonathletes the differences were found in 5JT (*p* = 0.01), 5 m (*p* = 0.02), 10 m (*p* = 0.01), and 20 m (*p* = 0.01) tests. Additionally, a significant interaction of gender and biological maturity was detected for CMJ (*p* = 0.03), CMJA (*p* = 0.01), and Zig Zag (*p* = 0.05) in athletes. The findings of the current study confirm the importance of maturity status in the assessment of physical performance. As a consequence, a more rational selection of talented athletes could be provided, also enabling the timely development of physical performance in nonathletes as a “window of opportunity”.

## 1. Introduction

During the process of maturation, major changes occur in the organism of a child, from anthropometric to functional ones [1]. Most physical abilities have their own growth pattern that improves during childhood as a function of physical growth [2,3]. It is a known fact that growth during childhood is non-linear with a rapid increase in body weight and muscle mass after the peak height velocity (PHV) [2]. Moreover, changes occurring with biological maturation include increased muscle strength and power [4]. However, these increases are only partly due to increases in body mass, which should be taken account [5].

Maturation assessment can be conducted by evaluating skeletal development and secondary sexual characteristics [6]. Mirwald, Baxter-Jones, Bailey, and Beunen [7] confirmed the reliability of a non-invasive method that evaluated the time of PHV, which made a maturation-related categorization possible [8,9]. The necessity of grouping children based on PHV status comes from the necessity to use the periods of development more appropriate for the development of certain physical performance [10].

The difference between biological and chronological maturity is important, making those with early maturation more physically dominant and granting more chances in sports selections [11]. The intentional selection of these children for a position in the team represents a risk, since the children who have advanced in the sense of biological maturity and demonstrate superior physical performance in early adolescence may not retain these physical advantages later in adolescence and adult age [12]. In most sports, more biologically mature children have superior anthropometric characteristics and are dominant in tasks requiring speed, power, and strength [11,13]. However, Figueiredo, Coelho-e-Silva, and Malina [14] found that more biologically mature children did not always show better results in specific motoric tests, although they passed the selection as more talented than their age-mates.

Athletes who reach PHV earlier compared to nonathletes [15] demonstrate better physical performance, despite the fact that training does not have a significant impact on growth and maturation [16]. Additionally, Murtagh, Brownlee, O’Boyle, Morgans, Drust, and Erskine (2018) found out that athletes have better results in speed and explosive power tests in almost all PHV groups compared to nonathletes. Numerous studies demonstrated that physical performance changes with the process of maturation [2,3] and that participation in sports can improve them [17,18]. However, it is not known whether certain maturation periods are more appropriate for the development of particular physical performance. Moreover, the studies that have investigated the differences within groups of athletes and nonathletes taking into account the maturation status and gender are rather scarce. Therefore, the aim of the study was to establish the effects of maturation on physical performance in both boys and girls, athletes and nonathletes.

## 2. Materials and Methods

### 2.1. Participants

In total, 231 subjects participated in the study, both athletes and nonathletes of either gender. There were 131 athletes distributed in 6 groups according to their biological maturity and gender: PrePHV boys (*n* = 27, chronological age: Mean ± SD 12.9 ± 0.7), PrePHV girls (*n* = 8, chronological age: Mean ± SD 10.6 ± 0.7), MidPHV boys (*n* = 12, chronological age: Mean ± SD 12.9 ± 0.5), MidPHV girls (*n* = 10, chronological age: Mean ± SD 11.6 ± 0.5), PostPHV boys (*n* = 33, chronological age: Mean ± SD 14.8 ± 0.9), and PostPHV girls (*n* = 41, chronological age: Mean ± SD 13.7 ± 1.2).

One hundred nonathletes were also distributed in six groups as to their biological maturity and gender: PrePHV boys (*n* = 9, chronological age: Mean ± SD 13.1 ± 0.3), PrePHV girls (*n* = 9, chronological age: Mean ± SD 11.4 ± 0.2), MidPHV boys (*n* = 17, chronological age: Mean ± SD 13.6 ± 0.7), MidPHV girls (*n* = 25, chronological age: Mean ± SD 11.9 ± 0.5), PostPHV boys (*n* = 21, chronological age: Mean ± SD 14.5 ± 0.4), and PostPHV girls (*n* = 19, chronological age: Mean ± SD 12.5 ± 0.3). The descriptive parameters of athletes and nonathletes can be seen in Table 1 and Table 2. In the selection of athletes, the criterion was applied according to which an athlete is a child who is an active member of a sports club. Moreover, the criteria for the athletes were that they have performed sports activities for at least 8 h a week for the past two years. Inclusion criteria for nonathletes included <2 h per week of physical activities. Participants were excluded from the study if they had any injures in the past 6 mounts. All procedures were approved by the ethical committee at the Faculty of Sport and Physical Education, University of Nis, and in accordance with the Declaration of Helsinki. Parent/guardian consent was obtained for all the participants, since they were all below 18 years of age at the time of the study.

### 2.2. Procedures

#### 2.2.1. Anthropometric Characteristics

Anthropometric characteristics were measured with participants barefoot and with light sports equipment [19]. Body mass (kg) measurements were performed on an electronic scale, with 0.1 kg accuracy (Omron BF 511, Kyoto, Japan); body height, sitting height, and leg length measurements were performed using an anthropometer with 0.1 cm accuracy (the anthropometer by Martin). Body mass index (BMI) was calculated as the ratio of body mass in kilograms and squared body height in meters (kg/m^2^).

#### 2.2.2. Maturation Assessment

Biological maturity was calculated for each participant using the formula established by Mirwald, Baxter-Jones, Bailey, and Beunen [7]. It is a non-invasive method that evaluates the time of greatest increase in height (PHV) taking into account anthropometric characteristics (body height, sitting height, leg length) and chronological age. Based on the maturity offset value (in years), the value was obtained (expressed in years) indicating the time passed since PHV; then the value of maturity offset was subtracted from the chronological age, obtaining the exact years when PHV was reached (Age@PHV). The maturity offset formula for boys and girls was as follows:

GIRLS: Maturity offset (years) = −9.376 + (0.0001882 × [Leg Length × Sitting Height]) + (0.0022× [Age × Leg Length]) + (0.005841 × [Age × Sitting Height]) − (0.002658 × [Age × Weight]) + (0.07693 × [weight: height × 100])

BOYS: Maturity offset (years) = −9.236 + (0.0002708 × [Leg Length × Sitting Height]) + (−0.001663 × [Age × Leg Length]) + (0.007216 × [Age × Sitting Height]) + (0.02292 × [weight: height × 100])

The dividing of the participants into PrePHV, MidPHV, and PostPHV groups was performed according to the Maturity offset. The participants whose Maturity offset value ranged from −0.5 to 0.5 were assigned to the MidPHV group; those with a Maturity offset below −0.5 were assigned to PrePHV; and those with a Maturity offset above 0.5 were assigned to the PostPHV group [20].

### 2.3. Testing Protocol

The warm-up protocol was applied after anthropometric measurements—a standard warm-up was performed for 10 min and consisted of running (40–60% of HRmax) and lower extremity dynamic stretching exercises.

#### 2.3.1. Assessment of Explosive Power

The assessment of explosive power was performed using four tests in a sports hall on a flat surface, with a minimal impact of external factors. The three tests for the assessment of vertical jumping ability were CMJ, CMJA, and SJ. They were performed in accordance with the already-described protocols [21]. These tests are valid and reliable to measure explosive power [22]. Vertical jumping ability was evaluated using the system of photoelectric cells (Optojump, Microgate, Bolzano, Italy), which demonstrated validity and reliability in the assessment of such jumps [23]. Horizontal jumping ability was assessed using 5JT, according to the protocol described by Chamari, Chaouachi, Hambli, Kaouech, Wisloff, and Castagna [24], who had confirmed its validity and reliability.

When performing a CMJ, the arms of the examinee were isolated in a position on the hips, so that arm swings could not influence the ability tested by the jump. The examinee stood upright for a few seconds, descended, and assumed the semi-squat position with a 90° angle between the thigh and lower leg, and without stopping at the point of movement direction change, made use of the elastic energy, performed his maximal vertical jump, and then landed with slight knee flexion. The protocol of CMJA is identical to that for CMJ, with a different arm position—the hands are free for movement and in the function of a swing in order to achieve maximum jump height. The squat jump is a jump from a static position. The legs are in 90° knee flexion; the arms are isolated on the hips in order not to influence the ability tested by the jump. After a still phase in the full squat position, a maximum vertical jump is performed, landing with slight flexion at the knees. The purpose of the test is to evaluate the concentric component during the performance of the jump. The 5JT test consists of 5 consecutive jumps with joined feet at the beginning and end of the test. From the starting position, consecutive jumps are performed with the left and right legs, and after the 4th jump, the last jump is performed with joined feet landing. Horizontal jumping ability is measured with a measuring tape from the front foot edge in the starting position to the back foot edge in the final position.

#### 2.3.2. Speed Assessment

The speed assessment was performed using 5 m, 10 m, and 20 m sprint tests according to the described protocol [25]. The participants performed three repetitions with 120 s pauses in between, with the fastest performance taken for statistical analysis. The system of photoelectric cells (Witty System, Microgate, Bolzano, Italy) was placed on the start line and at distances of 5 m, 10 m, and 20 m from the start. Lap times were thus recorded, as well as the increases in speed. The validity and reliability of speed measurement using photocells were proven by the paper by Zabaloy, Freitas, Carlos-Vivas, Giraldez, Loturco, Pareja-Blanco, Galvez Gonzales, and Alearaz [26]. The system of photocells was placed at approximately hip height for all participants to assure that the gates were passed through with only one part of the body [27]. The test involved running at a maximum speed for a distance of 20 m; the participants assumed the standing start position at 50 cm from the start line and were instructed to cross the finish line as fast as possible.

#### 2.3.3. Change of Direction Speed (CODS) Assessment

CODS was assessed using the T-test, Zig Zag, and Slalom tests, following the accepted protocols [28,29]. The validity and reliability of these tests were determined by Sporiš, Jukić, Milanović, and Vučetić [29]. The participants performed three repetitions of each test with 120 s pauses in between, and with 5 min pauses between different CODS tests; the fastest performance was taken for further analysis. Measurements were performed using the system of photoelectric cells (Witty System, Microgate, Bolzano, Italy), and the equipment was placed in accordance with the recommendations by relevant authors [27]. 

The T-test is a CODS test involving the combination of movement: Forward, lateral, and backward. Four cones are placed in the shape of the letter “T”, two cones in a line (10 yards apart) and one on each side of the end of the line. The examinee assumes the standing start position 50 cm from the start line and is instructed to cross the finish line as fast as possible. He runs forward to the intersection of the side lines and touches the cone with the right hand, continues laterally to the left and touches the cone with the left hand, continues laterally to the right and touches the cone with the right hand, continues laterally to the left to the intersection of the side lines and touches the cone with the left hand, and continues backward to the finish/start line. The Zig-Zag test requires the examinee to run the planned course in the shortest time possible. The test consists of four cones placed at the corners of a rectangle (10 feet × 16 feet), with another cone placed at the center. If the cones placed at the corners are marked 1 to 4, starting from the longer side, and the central one is marked C, the test starts at cone 1, and continues running around the cones to C, 2, 3, C, 4, and back to 1. The participants assumed the standing start position with both legs behind the start line. Six cones were placed at a 2 m distance, with the first cone placed 1m from the start line. Each examinee stood facing the start line, with legs apart. After the signal, participants ran from the first cone to the second cone, moving around it with the right side of the body. Then they continued running, alternating left and right around the cones, until reaching the last cone. They then turned around and continued slaloming to the finish/start line.

### 2.4. Statistical Analysis

The data were analyzed using IBM SPSS (version 24.0; Inc., Chicago, IL, USA). The Kolmogorov–Smirnov test was applied and the assumption for normal distribution was confirmed. The mean and standard deviation (SD) were calculated for all variables. A 2 × 3 (gender × PHV group) mixed factor analysis of variance (ANOVA) was used to analyze the effects of gender, maturation stage, and their interaction for CMJ, CMJA, SJ, 5 mJT, 5 m sprint, 10 m sprint, 20 m sprint, T-test, Zig Zag, and Slalom tests. The Tukey post hoc test was used to examine the differences between PHV groups. Moreover, an analysis of covariance (ANCOVA) was used to determine the influence of the covariates, chronological age, body mass, and BMI on physical performance. The a priori alpha was set at 0.05.

## 3. Results

Table 3 and Table 4 present the results of the 2 × 3 ANOVA, describing the differences and interaction effects between the genders and PHV groups (Pre, Mid, and Post) in athletes (Table 3) and nonathletes (Table 4) in physical performance tests (CMJ, CMJA, SJ, 5JT, 5 m, 10 m, 20 m *t*-test, Zig Zag, Slalom). A significant interaction of gender and biological maturity was noted for CMJ (*p* = 0.03; η2 = 0.06), CMJA (*p* = 0.01; η2 = 0.1), and Zig Zag (*p* = 0.05; η2 = 0.05) tests among athletes. There were no interaction effects among nonathletes. Additionally, significant differences between the participants of different maturation stages were seen in athletes for SJ, 5JT, T-test, and 10 and 20 m sprints (*p* < 0.05) (Table 3). Post-hoc analysis (Tukey test) indicated that PostPHV participants had better results in all tests of explosive power (CMJ, SJ, 5JT), compared to Pre- and MidPHV participants. In speed tests (10 m and 20 m) and CODS tests (T-test and Zig Zag), PostPHV participants performed better compared to MidPHV participants. Significant differences in average values between boys and girls were found in all variables except in 5 m (*p* = 0.19), with boys having statistically significantly superior results.

Significant differences between participants in different maturation stages were found in nonathletes in the following variables: 5JT (*p* = 0.01), 5 m (*p* = 0.02), 10 m (*p* = 0.01), and 20 m (*p* = 0.01) (Table 4). Post-hoc analysis (Tukey test) indicated that PostHPV participants had better results in the explosive power test (5JT) and speed tests (5 m, 10 m, 20 m), compared to PrePHV participants. PostPHV participants were better in the 5 m test compared to the MidPHV group, while MidPHV participants were better in the 20 m test compared to the PrePHV group. Significant differences were found in the average test values between boys and girls in all tests, except in the Zig Zag (*p* = 0.06) and Slalom (*p* = 0.28) tests, with boys having statistically significantly better results.

ANCOVA was used to examine the influence of various factors on test results. When chronological age, weight, and BMI were selected as covariates, differences between athletes belonging to different maturation stages were found in CMJ (*p* = 0.01) and CMJA (*p* = 0.01) tests. The results were similar for nonathletes where differences exist in the CMJA (*p* = 0.03) and SJ (*p* = 0.04).

Differences between athletes belonging to different maturation stages exist in the Slalom test (0.02), when adjusted for chronological age. When participants’ weight and BMI were used as a covariate, differences were found in all tests except the 5 m test (weight *p* = 0.97; BMI *p* = 0.21). In nonathletes who belong to different maturation stages, there were no differences in tests when chronological age was used as a covariate. In addition, including weight and BMI as covariates affects the differences among nonathletes in all tests except the 5 m test (0.25) and the Slalom test (0.12) for weight and BMI, respectively.

## 4. Discussion

The aim of the study was to establish the role of maturation in the development of the physical performance of bot, boys and girls, athletes and nonathletes. The data were analyzed and presented separately for athletes and nonathletes, taking into account the factors of maturation and gender. The results showed that there were differences between the participants from different categories of biological maturation. Only the 5 m and Slalom tests did not show a significant difference in athletes, while in non-athletes, there is a significant difference in the 5JT, 5 m, 10 m, and 20 m tests. Significant differences in average test values among boys and girls were not found in only the 5 m test in athletes and in the Zig Zag and Slalom tests in nonathletes. Moreover, when controlling for age, body mass, and BMI, some significant differences regarding maturation stage remained in both athletes and nonathletes, especially in vertical jump tests (SJ, CMJ, and CMJA).

The physical growth of athletes and nonathletes occurs following the same sequence regardless of the differences in dynamics, tempo, and years when PHV is reached [30]. The differences occurring in adolescence in individuals of the same chronological age are those that describe biological maturation and are an essential factor of impact on growth and physical fitness. Therefore, this factor has an immense impact on the physical activity of adolescents [2,31]. The rate of physical growth is largely responsible for interindividual differences in the physical performance of adolescents [1]. With the sudden growth of adolescents (rapid increases in height and weight), there are variations in the time and speed of growth, which is associated with improved physical performance related to speed and power [11].

The results showed that body height and mass increase with higher maturity stages (Table 1 and Table 2). In addition, athletes in their PostHPV maturity status had significantly better results in the tests of vertical jumping ability and CODS compared to those from younger maturation groups. The differences could be explained by increased anthropometric characteristics, hormone levels, and muscle power caused by puberty, and these could have an impact on increased physical fitness in later maturation stages [32]. The results of our study agreed with these findings, showing that athletes (Table 3) from the PostPHV group had better results in all explosive power tests compared to the other two groups, and better results in speed tests (10 m and 20 m) and CODS tests (T-test and Zig Zag) compared to MidPHV participants. The results obtained for nonathletes (Table 4) showed that PostPHV participants were superior to PrePHV participants in the test of explosive power (5JT) and in all speed tests. PostPHV participants were better than MidPHV participants in the 5 m test, while MidPHV participants were better than PrePHV in the 20 m test. The observed differences were the consequence of differences in maturation stages, with the differences between the groups being even greater in athletes than in nonathletes. PostPHV athletes had better results than MidPHV and PrePHV groups in most of the tests, which was not the case with nonathletes. The reason behind such results was better utilization of sensitive phases in athletes, who had advanced more than nonathletes during these phases. The key period in the development of power starts with adolescence and continues throughout adulthood, primarily due to rapid increases in muscle power under the influence of biological maturation [33]. The effects of participation in sports on the development of speed depend on neuromuscular adaptation, which has been seen to be the principal factor. Maturation has an impact on this ability and the best periods in the development of speed are childhood and adolescence [10]. The results obtained by Meyers, Oliver, Hughes, Lloyd, and Cronin (2017) indicated that in pre-PHV individuals, speed was largely associated with step frequency, while in post-PHV individuals, speed was predominantly associated with step length. With increasing anthropometric characteristics and longitudinal dimensions, above all, step length is increased, which can contribute to greater running speeds. According to certain models of motor skill development, the best time for the development of CODS starts with prepuberty [10]. Regarding the best time for the development of physical performance, as a “window of opportunity”, the MidPHV period has often been reported for most physical performance, but for some abilities, it is better to develop in the PostPHV period [10]. That “window of opportunity” could be seen as the reason for the intergroup differences obtained, demonstrating that the number of statistically significant differences increased in more biologically mature participants.

In one similar study, young male athletes were taller and heavier compared to young female athletes and achieved better results in vertical jumping tests and CODS tests. The same study showed that female athletes were stronger and thinner than most of their female age-mates who do not participate in sports, but they do not achieve better results regarding height, power, and speed tests [32]. The results of our study agreed with this study, presenting the significant differences between male and female athletes (Table 3), in all variables except in the 5 m test, with boys having significantly better results. Significant differences were detected as well between nonathlete boys and girls (Table 4) in all tests, except in Zig Zag and Slalom, with boys having significantly better results. The results obtained by Lesinski, Schmelcher, Herz, Puta, Gabriel, Arampatzis, Laube, Busch, and Granacher (2020) indicated that the interaction of gender with biological maturity had a significant impact on increased differences in physical performance tests with increasing biological maturity and that the gender-related differences in physical fitness were rather small in early phases of biological maturity. The results of the current study corroborated the results of the aforementioned study, indicating a significant interaction of gender with biological maturity in athletes (Table 3) in explosive power tests (CMJ and CMJA) and CODS (Zig Zag). This interaction indicated increasing differences between boys and girls from early periods towards later phases of biological maturity. Although the changes in power, strength, and speed that occur under the impact of body growth are expected [2], the impact of physical activity on physical performance should be expected, since the advantage related to superior test results represents a decisive variable of success in both genders [34,35,36], and the difference in the trend of physical performance development should normally be expected in athletes in comparison to nonathletes.

The limitation of this study could perhaps refer to the difficulties in finding enough participants of this age involved in a programmed training process for longer periods of time, especially in the PrePHV phase of maturity. Another limitation may lie in the lack of uniformity of the groups to be compared, since it is not possible to determine in advance how many of the participants will belong to which PHV group. This was largely resolved by testing a larger total number of participants. Finally, we should consider the fact that a noninvasive method was used in this study, which, according to the formula, evaluates the time of greatest increase in height, taking into account anthropometric characteristics. It is assumed that radiography would be more precise in establishing the biological age of the participants and the obtained results would be more accurate, but it is also more expensive and involves exposure of the participants to ionizing radiation. Nevertheless, besides these limitations, the strength of the study lies in the fact that we have included both boy and girl athletes and nonathletes. Moreover, the athletes were from different sports compared to most studies that involved athletes mainly from one sport.

## 5. Conclusions

The results showed differences in biological maturity among athletes in physical performance. The results were similar in nonathletes, with PostPHV participants being dominant compared to other groups. Gender-related differences were found as well—boys were superior in most of the tests, regardless of their participation in sports. The findings indicate that the assessment of physical performance should be based on maturity status, since differences may be caused more by biological maturity than by chronological age. This can be an appropriate practice in the process of selection in various sports. Coaches can thus avoid the risks associated with investing in athletes in the process of biological maturation who are superior in physical performance tests solely on account of being in later phases of biological maturity. Future studies should focus on determining the most optimal criteria for the prediction of the maturity offset period, which would hopefully provide greater accuracy in the assessment of biological maturity and by which we would approach the gold standard for radiographic assessment of biological maturity.

## Figures and Tables

**Table 1:** Descriptive parameters of athletes.

Variables	Biological Age					
	PrePHV		MidPHV		PostPHV	
	Boys (*n* = 27)	Girls (*n* = 8)	Boys (*n* = 12)	Girls (*n* = 10)	Boys (*n* = 33)	Girls (*n* = 41)
	Mean ± SD	Mean ± SD	Mean ± SD	Mean ± SD	Mean ± SD	Mean ± SD
Chronological age	12.9 ± 0.7	10.6 ± 0.7	12.9 ± 0.5	11.6 ± 0.5	14.8 ± 0.9	13.7 ± 1.2
Maturity Age @ PHV	13.9 ± 0.6	11.7 ± 0.5	12.9 ± 0.5	11.5 ± 0.4	13.1 ± 0.7	12.1 ± 0.6
Maturity offset	−1 ± 0.4	−1.1 ± 0.7	−0.1 ± 0.2	0.1 ± 0.4	1.6 ± 0.8	1.7 ± 0.8
Sports experience (months)	37.9 ± 22.1	24.3 ± 15.5	43.7 ± 20.5	26.1 ± 18.9	46.6 ± 24.6	43.2 ± 26.7
Body height (cm)	157.7 ± 6.7	145.2 ± 8.2	169.3 ± 3.9	156.3 ± 5.2	179.2 ± 7.9	162.1 ± 5.5
Weight (kg)	52.2 ± 11.1	41.8 ± 10.4	65.2 ± 10.9	46.3 ± 5.9	75.1 ± 13.2	55.7 ± 7.9
BMI (kg/m^2^)	20.8 ± 3.5	19.8 ± 4.9	22.8 ± 4.2	18.9 ± 2.2	23.5 ± 4.5	21.2 ± 2.7

PrePHV = Pre Peak Height Velocity; MidPHV = Mid Peak Height Velocity; PostPHV = Post Peak Height Velocity.

**Table 2:** Descriptive parameters of nonathletes.

Variables	Biological Age					
	PrePHV		MidPHV		PostPHV	
	Boys (*n* = 9)	Girls (*n* = 9)	Boys (*n* = 17)	Girls (*n* = 25)	Boys (*n* = 21)	Girls (*n* = 19)
	Mean ± SD	Mean ± SD	Mean ± SD	Mean ± SD	Mean ± SD	Mean ± SD
Chronological age	13.1 ± 0.3	11.4 ± 0.2	13.6 ± 0.7	11.9 ± 0.5	14.5 ± 0.4	12.5 ± 0.3
Maturity Age @ PHV	14.1 ± 0.5	12.3 ± 0.4	13.6 ± 0.5	11.8 ± 0.4	13.3 ± 0.5	11.5 ± 0.4
Maturity offset	−1.1 ± 0.3	−0.9 ± 0.4	0.02 ± 0.22	0.1 ± 0.3	1.3 ± 0.6	1.1 ± 0.3
Sports experience (months)	0	0	0	0	0	0
Body height (cm)	156.6 ± 6.9	146.4 ± 3.8	167.1 ± 4.6	155.1 ± 3.8	175.6 ± 5.9	161.7 ± 5.5
Weight (kg)	42.5 ± 8.5	37.4 ± 5.9	61.9 ± 8.7	44.5 ± 5.9	68.3 ± 12.8	56.29 ± 7.9
BMI (kg/m^2^)	17.2 ± 2.9	17.4 ± 2.2	22.2 ± 3.1	18.5 ± 2.2	22.1 ± 3.5	21.7 ± 3.3

PrePHV = Pre Peak Height Velocity; MidPHV = Mid Peak Height Velocity; PostPHV = Post Peak Height Velocity.

**Table 3:** Differences in physical performance related to maturation stage and gender of young athletes.

Variables	Biological Age						Main/Interaction Effects								
	PrePHV		MidPHV		PostPHV		PHV			Gender			PHV × Gender		
	Boys	Girls	Boys	Girls	Boys	Girls	F	p	η2	F	p	η2	F	p	η2
	Mean ± SD	Mean ± SD	Mean ± SD	Mean ± SD	Mean ± SD	Mean ± SD									
CMJ (cm)	23.3 ± 4.2	22.2 ± 3.4	23.6 ± 3.6	21.1 ± 4	30.6 ± 7.1 ^†^*	23.9 ± 3.9 ^†^*	13.04	0.01	0.17	10.95	0.01	0.08	3.65	0.03	0.06
CMJA (cm)	28.6 ± 5.9	26.8 ± 5.3	27.9 ± 4.1	25.8 ± 4.1	37.5 ± 7.8 ^†^*	27.5 ± 4.3 ^†^*	11.67	0.01	0.16	14.64	0.01	0.11	6.76	0.01	0.1
SJ (cm)	22.8 ± 4.7	20.6 ± 2.6	22.8 ± 4.8	21.4 ± 3.9	26.5 ± 5.6 ^†^*	21.9 ± 4.1 ^†^*	3.79	0.03	0.06	7.8	0.01	0.06	1.29	0.28	0.02
5JT (cm)	8.7 ± 0.8	7.6 ± 0.8	8.9 ± 0.8	8.1 ± 0.8	10.1 ± 1.2 ^†^*	8.8 ± 0.8 ^†^*	19.92	0.01	0.24	31.99	0.01	0.2	0.39	0.68	0.01
5 m (s)	1.2 ± 0.1	1.2 ± 0.2	1.2 ± 0.1	1.3 ± 0.1	1.2 ± 0.1	1.2 ± 0.1	1.13	0.33	0.02	1.73	0.19	0.01	2.38	0.1	0.04
10 m (s)	2.1 ± 0.2	2.1 ± 0.2	2.1 ± 0.2	2.2 ± 0.1	1.9 ± 0.1 *	2.1 ± 0.1 *	3.83	0.02	0.06	7.72	0.01	0.06	0.99	0.37	0.02
20 m (s)	3.6 ± 0.3	3.9 ± 0.4	3.6 ± 0.3	4.1 ± 0.2	3.4 ± 0.2 *	3.7 ± 0.2 *	11.31	0.01	0.15	39.16	0.01	0.24	0.75	0.48	0.01
T-test (s)	11.9 ± 1.1	13.1 ± 0.7	11.7 ± 0.6	13.1 ± 0.9	11.3 ± 0.9 *	12.1 ± 0.9 *	9.19	0.01	0.13	29.76	0.01	0.19	1.08	0.34	0.02
Zig Zag (s)	7.2 ± 0.4	8.1 ± 0.6	7.3 ± 0.5	8.2 ± 0.5	7.1 ± 0.5 *	7.5 ± 0.6 *	7.22	0.01	0.1	42.88	0.01	0.26	3.17	0.05	0.05
Slalom (s)	8.5 ± 0.8	9.1 ± 0.6	8.8 ± 0.7	9.3 ± 0.4	8.6 ± 0.7	8.8 ± 0.6	2.78	0.07	0.04	8.17	0.01	0.06	0.82	0.44	0.01

SD = standard deviation; F = F test results; ^†^ *p* < 0.05 vs. PrePHV; * *p* < 0.05 vs. MidPHV; ^○^ *p* < 0.05 vs. PostPHV; CMJ = countermovement jump; CMJA = countermovement jump with free arms; SJ = squat jump; 5JT = five-jump test.

**Table 4:** Differences in physical performance related to maturation stage and gender of young nonathletes.

Variables	Biological Age						Main/Interaction Effects								
	PrePHV		MidPHV		PostPHV		PHV			Gender			PHV × Gender		
	Boys	Girls	Boys	Girls	Boys	Girls	F	p	η2	F	p	η2	F	p	η2
	Mean ± SD	Mean ± SD	Mean ± SD	Mean ± SD	Mean ± SD	Mean ± SD									
CMJ (cm)	22.2 ± 6.7	18.1 ± 4.1	24.2 ± 5.8	20.1 ± 3.5	26.1 ± 4.9	19.4 ± 3.8	1.88	0.16	0.04	24.24	0.01	0.21	0.89	0.41	0.02
CMJA (cm)	27.4 ± 6.9	21.5 ± 4.1	29.1 ± 7.2	23.4 ± 4.4	30.1 ± 5.9	22.2 ± 5.1	0.73	0.49	0.02	28.27	0.01	0.23	0.43	0.65	0.01
SJ (cm)	21.7 ± 6.8	17.1 ± 3.3	22.9 ± 6.4	18.8 ± 4.7	23.7 ± 4.2	17.1 ± 4.1	0.55	0.58	0.01	23.44	0.01	0.2	0.78	0.46	0.02
5JT (cm)	7.9 ± 1.2	6.6 ± 0.9	8.3 ± 0.8	7.3 ± 0.6	8.9 ± 1.1 ^†^	7.4 ± 1 ^†^	6.62	0.01	0.12	38.84	0.01	0.29	0.83	0.44	0.02
5 m (s)	1.2 ± 0.2	1.4 ± 0.2	1.2 ± 0.1	1.4 ± 0.2	1.1 ± 0.1 ^†^*	1.3 ± 0.1 ^†^*	4.42	0.02	0.09	46.56	0.01	0.33	0.32	0.73	0.01
10 m (s)	2.2 ± 0.3	2.4 ± 0.2	2.1 ± 0.2	2.4 ± 0.2	2.1 ± 0.1 ^†^	2.3 ± 0.2 ^†^	5.01	0.01	0.09	49.35	0.01	0.34	0.08	0.93	0.01
20 m (s)	4.1 ± 0.8	4.3 ± 0.3	3.7 ± 0.3 ^†^	4.1 ± 0.3 ^†^	3.6 ± 0.2 ^†^	4.1 ± 0.3 ^†^	4.89	0.01	0.09	28.2	0.01	0.23	0.49	0.62	0.01
T-test (s)	14.5 ± 5.6	15.9 ± 2.1	13.2 ± 1.2	14.7 ± 1.4	12.9 ± 1.1	14.8 ± 1.6	2.6	0.08	0.05	12.12	0.01	0.11	0.14	0.87	0.01
Zig Zag (s)	8.9 ± 2.5	9.1 ± 0.7	8.4 ± 0.7	8.8 ± 0.8	8.4 ± 0.7	9.2 ± 1.1	0.77	0.46	0.02	3.64	0.06	0.04	0.7	0.49	0.02
Slalom (s)	10.1 ± 3.4	10.1 ± 0.9	9.8 ± 0.8	9.9 ± 0.8	9.6 ± 0.6	10.4 ± 1.3	0.16	0.85	0.01	1.16	0.28	0.01	0.91	0.41	0.02

SD = standard deviation; F = F test results; ^†^ *p* < 0.05 vs. PrePHV; * *p* < 0.05 vs. MidPHV; ^○^ *p* < 0.05 vs. PostPHV CMJ = countermovement jump; CMJA = countermovement jump with free arms; SJ = squat jump; 5JT = five-jump test.

## Data Availability

The dataset used and/or analyzed during the current study is available from the corresponding author in response to a reasonable request.
